# The Feedback of the Chinese Learning Diagnosis System for Personalized Learning in Classrooms

**DOI:** 10.3389/fpsyg.2019.01751

**Published:** 2019-08-08

**Authors:** Xiaofeng You, Meijuan Li, Yue Xiao, Hongyun Liu

**Affiliations:** ^1^Faculty of Psychology, Beijing Normal University, Beijing, China; ^2^Collaborative Innovation Center of Assessment Toward Basic Education Quality, Beijing Normal University, Beijing, China; ^3^Beijing Academy of Educational Sciences, Beijing, China; ^4^Beijing Key Laboratory of Applied Experimental Psychology, National Demonstration Center for Experimental Psychology Education, Beijing Normal University, Beijing, China

**Keywords:** personalized learning, feedback, cognitive diagnosis, classroom, China

## Abstract

Personalized learning is tailored to each student’s strengths and needs. For personalized learning in the classroom, feedback is an essential part, which can provide useful guidance on how to improve learning and/or teaching. Based on the cognitive diagnosis theory, the Chinese Learning Diagnosis System (CLDS) offers timely feedback on student learning and teacher instruction. This study mainly describes the feedback of the CLDS in two parts. Part I introduces the feedback reports of the CLDS, and part II illustrates its application effectiveness in learning and teaching. Based on students’ mastery of an attribute, teachers can modify their teaching contents and schedules, and students have the opportunity to remedy their learning by themselves. As to the application effectiveness of the CLDS, the experiment results show that students enrolled in the experimental school with CLDS in 2012 had a significant improvement in their self-efficacy and achievement. Furthermore, most teachers pointed out that instructional time used for classroom unit tests was reduced by one third to one half, allowing them plenty of time to provide more detailed and individualized instructions to students.

## Introduction

Personalized learning, individualized instruction, personal learning environment, and direct instruction all refer to the efforts to tailor education to meet the different needs of students. The U.S. Department of Education’s National Educational Technology Plan (U.S. Department of Education, 2010) defined personalized learning as adjusting the pace of instruction (individualization), adjusting the instructional approach (differentiation), and connecting instruction to the learner’s interests and experiences. Culatta (2013), the former director of the Department of Education’s Office of Educational Technology, has noted that “Personalized learning may be the most important thing we can do to reimagine education in this country.” Typically, technology is used to try to facilitate personalized learning environments (Al-Zoube, 2009).

While all of the above definitions seem to refer to desirable goals in education, what does personalized learning really mean with respect to the classroom? Proponents of personalized learning say that many elements of curriculum, assessment, and instructional design must be presented in classrooms for students to succeed and that software systems are often used to manage and facilitate student-led instruction. Proponents believe that in personalized teaching, classroom learning activities have to be built upon students’ prior knowledge and that teachers need to allocate time for exercises. In addition, advocates argue that teachers must continuously assess student learning based on clearly defined standards and goals and that students’ input into the assessment process is integral (Herrington and Oliver, 2000; Lindgren and McDaniel, 2012).

For personalized learning in the classroom, feedback is an essential component of effective learning and teaching. Winne and Butler (1994) claimed that “feedback is information with which a learner can confirm, add to, overwrite, tune, or restructure information in memory” (p. 5740). Feedback to students helps narrow the gap between current understanding and performance and intended learning goal (Hattie and Timperley, 2007). It helps students (or teachers) detect and correct errors and misconceptions and provides guidance on how to improve their learning (or teaching). Bellon et al. (1991) believe that academic feedback is more strongly and consistently related to achievement than any other teaching behavior and that the relationship is consistent across grades, socioeconomic status, races, or school settings. To benefit students’ learning and teachers’ instruction, effective feedback needs to be constructive, meaningful, constant, and timely. Therefore, we need a reliable and valid technique of measurement design and modeling.

In the studies of Kluger and DeNisi (1996) and Hattie and Timperley (2007), the feedback can operate at four levels, i.e., feedback about the task (FT), feedback about the processing of the task (FP), feedback about self-regulation (FR), and feedback about the self as a person (FS). Feedback at each level has different effectiveness. FT shows how well a task has been performed, and it is associated with correctness, neatness, behaviors, or other explicit criteria related to task accomplishment. Therefore, students focusing too much on FT may pursue an immediate goal rather than strategies to achieve the goal. Consequently, instead of making cognitive efforts, they will be eager to adopt trial-and-error approaches to develop informal hypotheses on the relationship among the instruction, the feedback, and the intended learning. FP, more specifically, focuses on the process of completing tasks, and it relates to strategies used by students for error detection. Therefore, this process-level feedback is more effective than FT in promoting deeper learning (Balzer et al., 1989). Unlike the outcome-oriented feedback, the process-level feedback focuses more on the goal setting process, and it is a direct and efficient way of shaping the individual’s solution to the task (Earley et al., 1990; Harks et al., 2014). FR indicates students’ process of self-monitoring and self-directing toward success, and it plays an important role in self-assessment and willingness to receive feedback. FS covers little information about the task. Instead, it focuses on the characteristics of the learner. Examples of FS include “You are a good girl” or “Well done.” To sum up, FS is ineffectual in enhancing learning, while both FR and FP are highly effective in deep processing and mastery of tasks (Hattie and Timperley, 2007). FT exhibits positive effects when the task-related information can subsequently help improve strategy processing or strengthen self-regulation, which too rarely occurs in practice yet (Hattie and Timperley, 2007).

The application of cognitive diagnostic theory in education promotes the technological development of FP. Cognitive diagnosis theory is considered as a core element of the theory of psychoeducational measurement for a new generation of tests. The objective of cognitive diagnosis is to provide more information about test takers’ mastery levels of each attribute and, therefore, allow an investigation of the cognitive processes, contents, and knowledge hidden inside their performance. Specifically, cognitive diagnosis can establish a relationship between observed test scores and cognitive characteristics of examinees, based on which the cognitive strategies they use for solving problems can be understood. In addition, it can classify examinees according to their mastery of required skills in the test in the field of education, enabling students to be informed about the strengths and weaknesses of their problem-solving strategies and skills (Tatsuoka, 1983, 1995). Therefore, cognitive diagnosis highlights how students can improve their performance and where teachers can pay more attention to students. In addition, it may help teachers learn more about their students and provide individualized instruction for students, thus guiding teachers to introduce personalized learning into classrooms.

Feedback should be used to answer questions about teaching effectiveness while learning activities are in progress, rather than at the end of an instructional unit or period (Layng et al., 2006). It is when we assign a value to assessment that we arrive at an evaluation or at the process of making judgments based on assessment and evidence (Levine, 2005). Therefore, feedback should be provided when the assessment is still fresh for students and teachers, and before the learning and teaching activities move on to the next step. Feedback should meet the individual’s needs, be linked to specific assessment criteria, and be received in time to benefit subsequent work. Feedback is valuable when received, understood, and acted on. However, how students and teachers analyze, discuss, and respond to feedback is as important as the quality of the feedback itself (Nicol, 2010).

The Chinese Learning Diagnosis System (CLDS^1^) developed by Biyouxue, a Chinese educational evaluation company, adopts the cognitive diagnosis theory to offer timely feedback on student learning and teacher instruction. It has successfully incorporated educational measurement into the teaching processes. Teachers assign tasks to students in a class. After completed, the assignments are submitted by the students and uploaded to a cloud server via a high-speed scanner. Then, one can analyze the degree of each student’s mastery of attributes based on the cognitive diagnosis theory by cloud computing. Next, feedback messages will be sent to students so that students can learn their strengths and weaknesses in each attribute. Teachers may also provide students with targeted teaching, depending on the types of attributes students have mastered. In this way, both instruction quality and student performance can be effectively improved. Thus, “individualized teaching” becomes more reliable when educational measurement theory and big data technology are integrated and applied in the classroom.

When it comes to personalized learning and individualized teaching, feedback “is seen as the key to moving learning and teaching forward” (Stobart, 2008). The CLDS based on cognitive diagnosis theory has the superiority of providing process-level feedback, which relates attributes and skills specified to students’ performance. In other words, CLDS can specify the content areas that need remedial instruction. Accordingly, this study mainly describes the feedback of the CLDS in two parts. Part I introduces the feedback report provided by the CLDS. Part II illustrates the effectiveness of CLDS in learning and teaching applications.

## The Clds

The CLDS aims to provide a set of effective measurement tools by establishing a “timely feedback system.” These measurement tools are designed based on cognitive attributes and the relationships between these attributes, while test data are analyzed using the cognitive diagnosis theory to obtain the mastery probability of each attribute for every student and the overall performance of the whole class on the attribute. Based on the mastery levels of students, teachers can modify the course content and schedules. Students can also remedy their weaknesses based on feedbacks. As a result, an environment for individualized teaching and personalized learning is created for teachers and students, which helps to improve both the quality of instruction and students’ performance. For a more specific description of the cognitive diagnosis model and the implementation process of the CLDS, please refer to You et al. (2017).

### Cognitive Diagnosis Models (CDMs) and Attributes Identification

#### CDMs

The purpose of cognitive diagnosis analysis is to identify which attributes students have mastered. Although many CDMs have been proposed, the deterministic inputs, noisy and gate (DINA) model is highly preferred by researchers because of its simple interpretation, good model–data fit, and relatively better classification accuracy compared with the general model when the sample size is small (de la Torre and Douglas, 2004, 2008; Henson et al., 2009; DeCarlo, 2012). In order to develop a more tractable polytomous CDM, DINA (Haertel, 1989; Junker and Sijtsma, 2001), which is much simpler and in widespread use (DeCarlo, 2011), is selected to be generalized for graded data (called the P-DINA, or the DINA-GD model). For each student, the mastery profile will be translated into a vector: α_𝐢_ = (*α*_*i*1_,*α*_*i*2_,…,*α*_*ik*_)′, where α_*ik*_=1 indicates that the *i* th student masters the *k* th attribute and otherwise α_*ik*_=0; *K* is the number of attributes. The DINA-GD model is described in the following equations, and the DINA model for dichotomous data is a special case of it.

The cumulative category response function of polytomously scored DINA model with *M_j_+1* categories is expressed as

(1) P(Xij≥m|α𝐢)={1-sj⁢m i⁢f⁢ηi⁢j≥mgj⁢m i⁢f⁢ηi⁢j<m (m=1,2,…,M)j

where X_ij_ is the observed response of student *i* to item *j*, α_𝐢_ represents the column vector of knowledge state for student *i* with components of α_*ik*_ (which equals either 0 or 1), and $\eta_{i\,j}=\frac{\alpha_{i}\,q_{j}^{\prime }}{q_{j}\,q_{j}^{\prime }}\times M_{j}$, in which *M*_*j*_ is the possible maximum graded score of item *j*, ${\text{𝐪}}_{\text{𝐣}}^{\prime }=\left(q_{j\,1},\ldots \,q_{j\,k}\right)$ indicates whether the *k* th skill is required to correctly answer item *j.* The part $\frac{{\text{α}}_{\text{𝐢}}\,{\text{𝐪}}_{\text{𝐣}}^{\prime }}{{\text{𝐪}}_{\text{𝐣}}\,{\text{𝐪}}_{\text{𝐣}}^{\prime }}$ represents the ratio of the required attributes possessed by student *i* for item *j*. That is to say, student *i* will exhibit a higher latent response η_*ij*_ if he or she masters more attributes required by item *j*. The possible values of η_*ij*_ are between 0 and 1. The slipping parameters for the ordered category *m* (*m* = 1, 2,…, *M*_*j*_) is defined as *s*_*jm*_ = P(*X*_*ij*_ < *m*|*η*_*ij*_≥*m*). The guessing parameters are defined as *g*_*jm*_ = P(*X*_*ij*_≥*m*|*η*_*ij*_ < *m*). The model assumes the order constraint of *s*_*jm*_≤*s*_*j*,*m* + 1_ and *g*_*jm*_≥*g*_*j*,*m* + 1_, which means that individuals are more likely to slip and less likely to guess correctly for the higher category of item *j*. The dichotomous latent response variable in DINA model is a special case of the polytomous latent response, where *M*_*j*_ = 1.

Then, the item response function will be:

(2) $$P\left(X_{i\,j}=m|{\text{α}}_{\text{𝐢}}\right)=\left\{ \begin{array}{cc} P\left(X_{\text{ij}}\geq m|{\text{α}}_{\text{𝐢}}\right)-P\left(X_{i\,j}\geq m+1|{\text{α}}_{\text{𝐢}}\right) & m=0,1,\ldots ,M_{j}-1\\ P\left(X_{\text{ij}}\geq m|{\text{α}}_{\text{𝐢}}\right) & m=M_{j} \end{array}\right.$$

*P*(*X*_ij_ = *m*|α_𝐢_) is the probability of student *i*, with α_𝐢_ obtaining score *m* for item *j*. Clearly, $\sum_{m=1}^{M_{j}}P\left(X_{i\,j}=m|{\text{α}}_{\text{𝐢}}\right)=1$.

Assuming local independence among items and students, the joint likelihood function of the DINA model for polytomous response is

(3) $$L\left(\text{𝐗}|\text{α}\right)=\prod_{i=1}^{N}L\left({\text{𝐗}}_{\text{𝐢}}|{\text{α}}_{\text{𝐥}}\right)=\prod_{i=1}^{N}\prod_{j=1}^{J}\prod_{m=1}^{M_{j}}P{\left(X_{i\,j}=m|\text{α}\right)}^{u_{i\,j\,m}}$$

$$\text{where}\;u_{\mathrm{ijm}}=\{\begin{array}{ll} 1 & ifX_{\mathrm{ij}}=m\\ 0 & \mathrm{otherwise} \end{array}$$

However, the joint maximum likelihood estimation which allows the simultaneous estimation of the model parameters and knowledge state vectors may lead to inconsistent parameter estimates (Baker, 1992). Instead of working with the conditional likelihood of *X* to obtain the model parameters, the maximization can involve the marginalized likelihood of the data.

(4) $$L\,\left(\text{𝐗}\right)=\prod_{i=1}^{N}L\,\left({\text{𝐗}}_{\text{𝐢}}\right)=\prod_{i=1}^{N}\sum_{l=1}^{L}L\,\left({\text{𝐗}}_{\text{𝐢}}|{\text{α}}_{\text{𝐥}}\right)\,p\,\left({\text{α}}_{\text{𝐥}}\right)$$

where *𝐋(𝐗*_𝐢_) is the marginalized likehood of the response vector of examinee *i*, *p*(α_𝐥_) is the prior probability of the skills vector α_𝐥_, and *L* = 2*^k^* (de la Torre, 2009).

In practice, the CLDS offers multiple-choice items and constructed-response items. Generally, most multiple-choice items are scored dichotomously, while constructed-response items are scored polytomously and yield graded response data with ordered categories. An expectation–maximization (EM) algorithm or an M-H (jumping M-H) algorithm and Markov chain Monte Carlo–Gibbs sampling (Tu et al., 2010, 2017) can be used to analyze the probability of a student’s mastery of each attribute (de la Torre, 2009). However, the EM algorithm is simpler.

#### Identifying Attributes and Constructing the Q-Matrix

**FIGURE 1:**
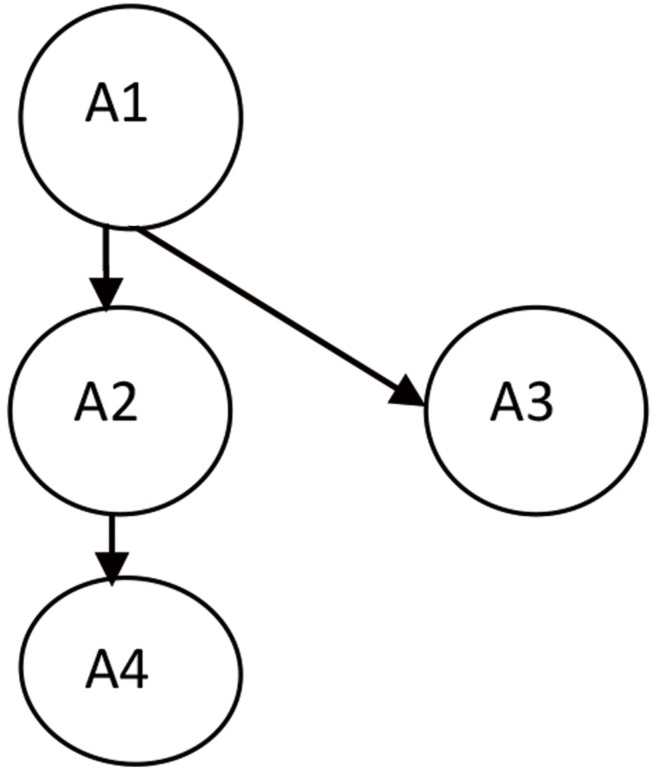
An example of the hierarchical relation between four cognitive attributes.

To identify attributes and construct of the Q-matrix, experts and teachers define the learning goals for each teaching unit according to curriculum standards and textbooks. Next, experts identify core skills and the relationship between these skills for each learning unit in accordance with the learning goals, that is, the cognitive attributes and the relationships between these attributes as specified in cognitive diagnosis theory. Finally, adjacency, reachability, incidence, and reduced-incidence matrices (Tatsuoka, 1983, 1995) of these attributes and their relationships are established to guide the item writing and test paper generation. For example, a given teaching unit may require teachers to examine four cognitive attributes: A1, A2, A3, and A4. The hierarchical relation between these four cognitive attributes is shown in Figure 1. Attribute A1 is a prerequisite for attribute A2, which means that a student must master A1 before learning A2. Similarly, a student must master A1 before learning A3, and master A1 and A2 before learning A4.

Following Tatsuoka’s method (Tatsuoka, 1983, 1995), the reduced-incidence matrix based on the four attributes and their relations shown above is shown as follows the reduced-incidence matrix based on the four attributes and their relations shown in Table 1 as follows.

**TABLE 1:** The reduced-incidence matrix of the four attributes in Figure 1.

	**Item**					
	**1**	**2**	**3**	**4**	**5**	**6**
A1	1	1	1	1	1	1
A2	0	0	1	1	1	1
A3	0	1	0	0	1	1
A4	0	0	0	1	0	1

This matrix means that with regard to the teaching unit, there must be at least six types of items examining different combinations of attribute mastery to diagnose students’ mastery of the four attributes. This design requires teachers using the CLDS to develop attribute-based items or to identify attributes for existing items. When the test is over, the system automatically feeds back the information about item parameters to the teacher. If the teacher feels that the Q-matrix needs to be modified, he or she can correct and resubmit it and then update the feedback report.

### Application Process of the CLDS

In order to describe the application process of the CLDS in detail, we use the first unit of the Chinese high school math curriculum, which is about “Set,” as an example.

(I) Test instrument development for the unit “Set.” For mathematics unit 1, students in grade 1 of the high school are required to recognize and understand the definition of the set, clearly identify the relationship between the set and its elements, understand what are union and intersection of two sets, and be able to find the union and intersection of two simple sets. Based on these teaching goals, the cognitive attributes of this unit are defined as (1) keeping in mind the definition of the set, (2) understanding the definition of the set, (3) understanding the representation of sets, (4) application of sets, (5) application of relationship between sets, (6) application of set calculation, and (7) application of set intersection. Teachers should create a test framework based on these cognitive attributes and develop an item bank.
(II) Test paper generation and cognitive attribute identification for each test item. Teachers design content specifications and determine the structure of a test paper, the type of items, and the number and score of every type of items according to the test framework and teaching goals. Twenty-two items have been developed, including 12 single-choice items, four fill-in-the-blank items, and six short-answer items. Scoring method and criteria are specified for each item. Then, the cognitive attribute examined by each item is identified accordingly.
(III) Collection of test information. Teachers are required to input the test paper information into the CLDS, and then the system generates an answer sheet automatically. Teachers need to download the answer sheet and print multiple copies of it before organizing the test. The CLDS integrates a multifunction digital machine (a printer capable of online data transmission) with cloud computing so that schools can collect data with low cost and high efficiency. A one-click scanning control program is installed for the multifunction digital machine, allowing it to fully play the role of scanning, photocopying, and printing. When the test is finished, the next step is to place each answer sheet onto the machine and scan and upload it to the cloud server of CLDS with just a click of the mouse. The server will sort out, identify, and process relevant work related to image documents. The cloud server will automatically review all objective items, while the subjective items will be reviewed online by teachers.
(IV) Data analysis. The DINA model (de la Torre, 2009; Tu et al., 2010) is used to analyze the attribute mastery model and obtain the mastery probability of each attribute for every student. Afterward, the average mastery probability of each attribute across students in each class can be calculated, as well as the number of students who have mastered each attribute. These results will be provided to schools, teachers, and students.
(V) Feedback and remedy. Feedback reports featuring individualized teaching and personalized learning in CLDS are introduced below.

### Feedback Reports of the CLDS

Feedback reports are divided into four categories: school report, class report, student report, and parent report. (See http://www.biyouxue.com/doclist for more information about how to use these CLDS reports). Teachers focus on class diagnostic feedback reports during their teaching process, while student diagnostic feedback reports are mainly used to improve student learning. Reports related to classes (or teachers) and students are described below. All reports are dynamically presented online, helping both students and teachers interactively select and compare the information they need.

The selection of the critical value (probability) tends to affect the judgment of students’ mastery of each cognitive attribute. Researchers believe that the criteria for determining whether a student has mastered a cognitive attribute should be in line with those identified by teachers or experts after they consider the assessment purpose and the context. For example, for formative assessment, which is the main purpose of the CLDS, it may be more costly to misclassify nonmastery as mastery, and therefore, we may wish to use a cutoff greater than 50 (Bradshaw and Levy, 2019). In practice, if teachers or experts have high expectations for student achievement, the value should be set at 0.8 or higher, while it may be below 0.5 if the expectations are low. Therefore, the CLDS sends each student’s mastery probability of each attribute directly to him/her, giving teachers and students more accurate feedback. Finally, the average mastery probability of each attribute in every class is summarized based on the preceding analysis and sent back to schools, teachers, and students. However, how to accurately interpret these probabilities is a big challenge for stakeholders in the operational setting (Bradshaw and Levy, 2019). More training and guidance are needed to help people correctly understand these numbers.

#### Student Diagnostic Feedback Report

Thanks to the interactive online presentations of the feedback, students can click and review the key items indicated in the report as needed, check their strengths and weaknesses in the tested unit, and recognize the contents to be improved. A student diagnostic feedback report includes, but is not limited to, the following content.

**FIGURE 2:**
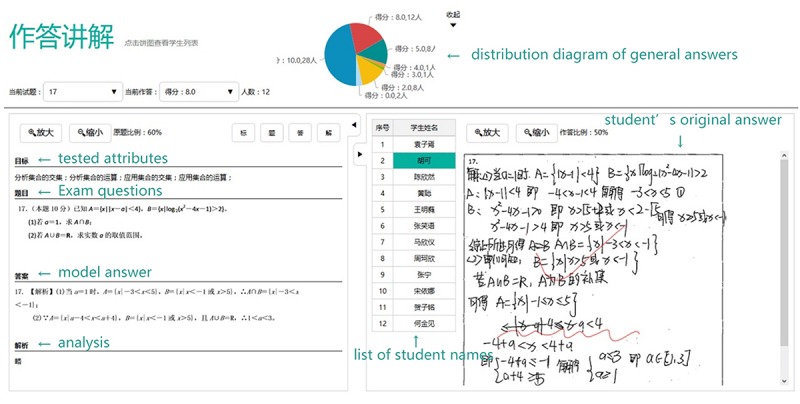
Student’s original answers and teacher’s comments.

**FIGURE 3:**
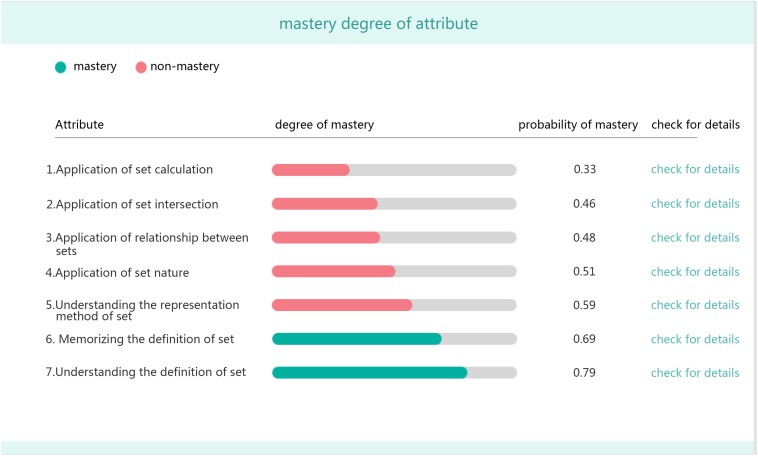
Student’s mastery probability of each attribute.

• Information related to exam items, including, but not limited to, the original items, correct answers, the overall performance of classmates, and the original answers of individual students as well as the teacher’s comments (Figure 2).

• Mastery probability of each attribute for the student (Figure 3).

• The strengths and weaknesses of the student in learning the unit.
• Personalized learning suggestions and the learning effect check.

The CLDS provides suggestions for individualized learning based on test results and types of mistakes made by the student. Afterward, learning results of the student can be evaluated through items with the same attributes chosen from an item bank.

• Providing an individualized item bank based on the student’s wrong answers.

Students who use the CLDS repeatedly can select the test scope and time scope where they are concerned. Then, for each of them, the system can generate an individualized item bank based on his/her wrong answers. In this way, the student can conduct targeted learning and periodic review, which prevents inefficient exercises and may also greatly improve self-efficacy.

#### Class (Teacher) Diagnostic Feedback Report

Teachers can check the following information through the CLDS: (1) the answer of each student to each item; (2) summary information of answers for all students in the class (or a group, e.g., all students below a certain score) to each item or items measuring a certain attribute; (3) a summary of the types of wrong answers that students give to a certain item; and (4) summary information of key items to be explained in detail. The general class (teacher) diagnostic feedback report consists of the following contents.

**FIGURE 4:**
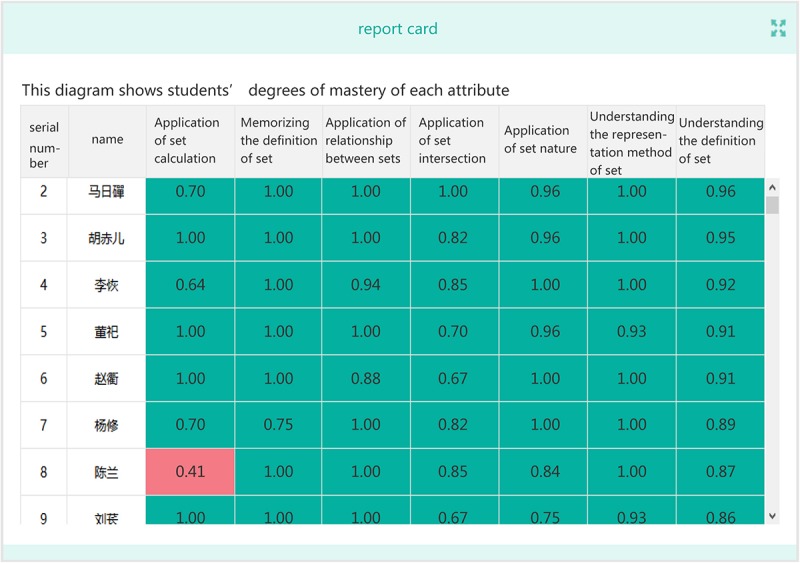
Student’s mastery of each attribute.

• General analysis of test results.
  This includes the mean and standard deviation of the test scores, as well as the difficulty and discrimination of each item.
• Students’ answers to each item.
  The CLDS can show the proportions of students choosing different options on objective items, as well as the percentage of students above or below a certain score. The answers given by particular students for a certain item can also be reviewed.
• The attribute mastery of students.
  The information on a student’s mastery level of each attribute (Figure 4) enables teachers to provide him/her with individualized instruction and targeted teaching.

• The average probability of attribute mastery at the class level.

**FIGURE 5:**
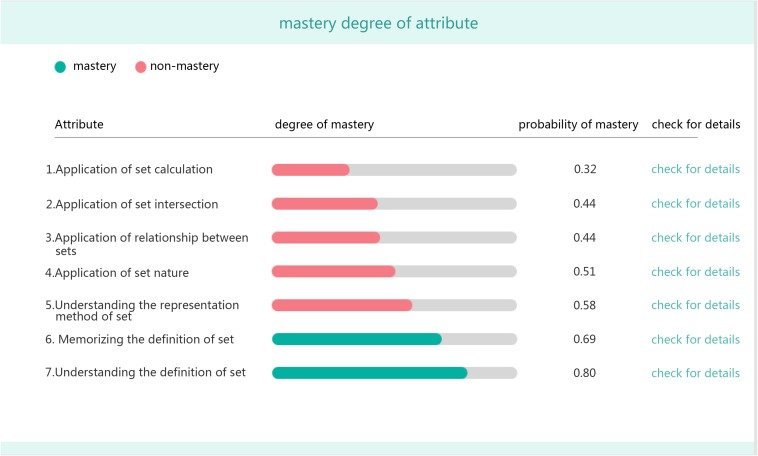
Feedback of class teaching efficiency of each attribute.

Based on the average probability of students’ mastery of each attribute, teachers can easily learn the general strengths and weaknesses of students, which refer to the attributes that most students have or have not mastered, respectively. Accordingly, teachers can provide more effective and efficient instructions in classroom teaching (Figure 5).

• A summary of different types of wrong answers:

For a specific test item, the CLDS will automatically summarize the types of wrong answers for students based on the teachers’ notes on the wrong answers made during their review of test papers. The summary will provide an overall picture of the wrong answers and help teachers provide targeted instruction. Teachers with experience in CLDS can choose items with accuracy rates below 60% in an interactive manner and create individualized teaching cases for key items as needed.

## Application Effectiveness of the CLDS

Since its online activation in 2012, the CLDS has been identified as one of the main products for teaching assessment and feedback in the Chinese classroom. Through targeted feedback, the CLDS provides support for the improvement of teachers’ instruction efficiency and the enhancement of students’ personalized learning. The section below describes the effectiveness of the CLDS using its application in an ordinary high school as an example.

### Experimental Design

In September 2013, the CLDS was introduced into school A and used to assist teachers with testing, assessment, and teaching. The college entrance examination scores of the students in this school have always been lower than the average scores of high schools in the city. The principal is faced with the problem that large classes can be challenging for teachers and can inhibit individualized instruction. In such cases, for satisfactory results, both teachers and students have to face regular homework and exams, which weigh them down. Some students are overwhelmed with excessive assignments from teachers and, thus, lose their learning motivation, which further lowers their overall self-efficacy.

A total of 3,139 students in this school were involved in the CLDS, including 1,255 liberal arts students and 1,884 science students. The CLDS recorded data from 204 tests for liberal arts students, including classroom tests, unit tests, interim tests, and final tests, which contain 6,015 items in all. It also recorded data from 205 such tests for science students, and each student answered 6,324 items. Meanwhile, the CLDS has generated 1,031 diagnostic feedback reports for the school, 5,673 reports for classes, and 309,239 reports for individual students. Also, there has been an average of 99 reports for each student. The study has been reviewed and approved by the Institutional Review Board of the Faculty of Psychology in Beijing Normal University and the committee in the local government. All parents of students signed written informed consent. Therefore, teachers, students, and parents had a clear understanding of this project and how the data were collected.

Based on the students’ mastery of attributes, teachers could modify their teaching contents and schedules. Specifically, they could find out the attributes that needed to be taught in class, as well as some attributes required by individual students. Also, teachers could provide targeted instruction according to types of wrong answers and the items with accuracy rates lower than a threshold (e.g., 60%). Based on the mistakes they made in a test, the level of mastery of each attribute, and the strengths and weaknesses within each unit, students had the opportunity to remedy their own learning. Further, the previous mistakes could prevent students from repeating aimless exercises and so achieve targeted learning.

In order to test the application effect of the CLDS, students enrolled in school A in 2012 were selected as the experimental group. Since students from school A and school B showed similar achievements, students from school B were taken as the reference group. School B, where the CLDS is not introduced into, has 396 students, including 209 liberal arts students and 187 science students. In this study, data of 547 students from school A were analyzed, including 211 liberal arts students and 336 science students. It is noted that liberal arts and science are two main academic streams of *gaokao* (i.e., the college entrance examination) in China. Liberal arts students take exams in Chinese, mathematics, English, politics, history, and geography, while science students take exams in Chinese, mathematics, English, physics, chemistry, and biology. For the 547 students from school A, the CLDS has generated 2,750 reports for classes and 60,170 reports for individual students.

### Data Collection and Analysis

Data collected in this study include pretest and posttest scores. The pretest scores are from the high school entrance examination in 2012, and the posttest scores are from the college entrance examination in 2015 after 3 years of high school study. As the entrance examinations for high schools or colleges in China both belong to the high-risk test category, a panel of experts was assembled and isolated to design an examination paper for each subject in full accordance with the “examination instruction.” The panel specified the exam framework, exam blueprints, and procedures of test paper design and review, pretest and analysis, and test paper generation. Based on the rigorous processes, those exams exhibited high reliability and validity.

In addition to students’ academic achievement, we also measured the academic motivation and self-efficacy of students from school A (which uses the CLDS) and school B (which did not use the CLDS from 2012 to 2015) through self-report questionnaires. The self-efficacy scale consists of 10 items regarding individuals’ self-perceptions of efficacy. These items are well suited for predicting how well individuals will perform. The items include, for example, “I think I will receive a good grade in this class” and “I am sure that I can do an excellent job on the problems and tasks assigned for this class” (Pintrich and De Groot, 1990; Mathieu and Martineau, 1997; Liang, 2000). The academic motivation scale contains 10 items concerning intrinsic motivation. The intrinsic motivation refers to the fact of doing an activity for itself, and the pleasure and satisfaction derived from participation. Those items include, for example, “Because I experience pleasure and satisfaction while learning new things” and “For the pleasure I experience when I discover new things never seen before” (Vallerand et al., 1992). Each item was measured on a 5-point Likert scale, anchored by 1 (strongly disagree) and 5 (strongly agree).

Confirmatory factor analysis (CFA) was used to validate the measurement models. The final model produced an acceptable fit to the data for each scale. For the self-efficacy scale, χ^2^ = 212.520, *df* = 35, *p* = 0.000, comparative fit index (CFI) = 0.961, Tucker–Lewis index (TLI) = 0.942, and root mean square error of approximation (RMSEA) = 0.094. For the academic motivation scale, χ^2^ = 229.348, *df* = 35, *p* = 0.000, CFI = 0.947, TLI = 0.932, and RMSEA = 0.090. The factor loadings of all items were high and substantial, whose values were between 0.638 and 0.821 (*p* < 0.001). Moreover, the omega coefficients of the academic motivation scale and the self-efficacy scale were 0.917 and 0.923, respectively, indicating high internal reliability for both scales (McNeish, 2018). Three experienced psychological experts examined the content validity of the two scales.

Data analysis was conducted using the SPSS 20.0 software. To explore the two schools’ performance in academic improvement, Cohen’s *d* value was calculated to compare the scores of the pretest and posttest.

### Effectiveness of the CLDS

In order to assess the efficiency of the CLDS, we analyzed the students’ self-efficacy and academic motivation before and after the use of CLDS in school A, as well as the self-efficacy and academic motivation of students from school B during the same period. Additionally, the pretest (high school entrance examination) and posttest (college entrance examination) scores of students in two schools were compared. College admission rates of school A before and after the use of CLDS were also collected, as well as the college admission rate of school B during the same period.

Table 2 presents the descriptive statistics of self-efficacy and academic motivation of students in two schools and Cohen’s *d* values with the corresponding standard errors for the differences between schools. It shows that student self-efficacy in school A improved significantly after the CLDS was applied (Cohen’s *d* of school *A* = 0.38, *p <* 0.001), while there were no obvious change in school B (Cohen’s *d* of school *B* = 0.09, *p >* 0.05). Additionally, the self-efficacy of students in school A were significantly lower than that in school B before the use of CLDS (Cohen’ *d* = −0.24, *p <* 0.001), whereas after using the CLDS, there was no significant difference between two schools (Cohen’ *d* = 0.05, *p >* 0.05). On the other hand, the academic motivation of students from school A and school B was improved. However, the gains in both schools were similar (Cohen’s *d* of school *A* = 0.33, *p <* 0.001; Cohen’s *d* of school *B* = 0.31, *p <* 0.001). Meanwhile, there was no significant difference of academic motivation between school A and school B neither before nor after the use of CLDS.

**TABLE 2:** Change of self-efficacy and academic motivation of school A and school B.

Variable	**School**	**Mean (SD)**		**Cohen’s *d* (SE)**
		**Before**	**After**	
Self-efficacy	School A	3.38(0.69)	3.65(0.72)	0.38(0.04)^∗∗∗^
	School B	3.54(0.62)	3.61(0.85)	0.09(0.05)
	Cohen’s *d* (SE)	−0.24(0.07)^∗∗∗^	0.05(0.07)	
Academic motivation	School A	3.55(0.50)	3.73(0.59)	0.33(0.04)^∗∗∗^
	School B	3.61(0.44)	3.79(0.67)	0.31(0.05)^∗∗∗^
	Cohen’s *d* (SE)	−0.13(0.07)	-0.11(0.07)	

*^∗∗∗^*p* < 0.001.*

Table 3 presents the descriptive statistics of academic achievement of liberal arts and science students from two schools, as well as the Cohen’s *d* values with standard errors for the differences between schools. It shows that there was no significant difference in the pretest (high school entrance exam) scores between schools, whether for liberal arts students or science students (Cohen’s *d* of liberal arts students = 0.05; Cohen’s *d* of science students = 0.04). However, the posttest (college entrance exam) average scores of school A were significantly higher than those of school B after 3 years of using the CLDS (Cohen’s *d* of humanities students = 0.31; Cohen’s *d* of science students = 0.66).

**TABLE 3:** Change in students’ academic achievement in school A and school B.

	**Mean (SD) of**		**Mean (SD) of**	
	**liberal arts students**		**science students**	
	**Pre-test**	**Post-test**	**Pre-test**	**Post-test**
School A	422.76(36.78)	469.48(66.95)	391.06(20.59)	478.22(62.39)
School B	421.25(27.11)	448.79(67.81)	390.00(32.14)	430.70(86.42)
Cohen’s d	0.05(0.10)	0.31(0.10)^∗∗∗^	0.04(0.09)	0.66(0.09)^∗∗∗^

*^∗∗∗^*p* < 0.001.*

Table 4 presents the college admission rates in school A and school B before and after the CLDS was used and the corresponding changes during the period. Although there was no difference in the pretest academic achievement between the two schools, the admission rates of tier 1 undergraduate and tier 2 undergraduate in school B were slightly higher than those in school A before the use of CLDS. After 3 years of using CLDS in school A, the admission rate of tier 1 undergraduates increased by 11.0% and that of tier 2 undergraduates by 47.2%. By contrast, the admission rate of tier 1 undergraduates in school B increased by only 4.1% and that of tier 2 undergraduates by only 0.8%. Thus, the results indicate that the growth ranges of admission rates of tier 1 and tier 2 undergraduates at school A are much higher than those at school B.

**TABLE 4:** Change in college admission rates of school A and school B.

**School**	**Category**	**College admission rate**		
		**Before**	**After**	**Change**
School A	Tier 1 undergraduate	1.9%	12.8%	11.0%
	Tier 2 undergraduate	17.4%	64.6%	47.2%
School B	Tier 1 undergraduate	4.8%	8.9%	4.1%
	Tier 2 undergraduate	26.5%	27.3%	0.8%

In addition to the above results, teachers also reported that the CLDS could benefit teaching and students’ independent learning. Most teachers indicated that the time for a unit test in the class had been reduced from the original 50 to 15–25 min. Thus, they had plenty of time to give more detailed instruction to individual students who made mistakes. In addition, students also reported that the individualized student feedback report helped them identify their own strengths and weaknesses. They could also figure out how to make appropriate solutions instead of comparing their exam scores with those of their classmates as they might do previously. The CLDS not only provides sufficient support for students’ learning and teachers’ instruction but also plays a positive and significant role for relevant people, especially parents. For example, some parents intend to provide learning instruction to their children by themselves. However, they had no knowledge of their children’s learning before, which might result in inappropriate instruction. Thanks to the CLDS, students’ learning progress becomes transparent to both teachers and parents. Therefore, both of them now can provide individualized instruction. Therefore, with the assistance of CLDS, the best results can be achieved with minimal efforts, whether from the perspective of students, teachers, or parents.

## Discussion

### The Improvement of Personalized Learning and Teaching Owing to the Feedback

The CLDS collects abundant data and information to achieve personalized feedback on learning and teaching. Specifically, CLDS records process data and responses of all exercises and tests in a complete manner via information and technology. Then, it analyses these data based on measurement theory and CDM. With such rich data, the CLDS not only provides more adequate evidence for exploring the general rules of learning but also establishes a foundation for adaptive testing and personalized learning. Unlike traditional testing models providing test scores only, data analysis based on CDM is more compatible with the concept of individualized testing feedback. Thus, teachers’ teaching activities and students’ solutions to their deficiencies can be more targeted and personalized according to the results of cognitive diagnosis analysis. The design concept of focusing on student performance greatly enhances the efficiency of teacher instruction and student learning.

The CLDS provides guidance for teachers’ instructional design and reflection by taking into account the characteristics of universality and individuality. Specifically, the CLDS can focus on core skills (attributes) in the course of achieving teaching goals and allows teachers to define their own core skills (attributes) for their curriculum design in the system. As requested, teachers can select three to nine core skills and describe them with a few words. This procedure requires teachers to further reflect on their teaching process and focus, which is helpful to establish the goals of both the teaching and performance assessment. If the teacher has clearly defined a few core skills, the diagnostic results are more targeted. Thus, the purpose of the current exercises can be easily explained to students.

With FP based on CDM, the CLDS also provides rich evidence for establishing students’ learning goals. Additionally, data accumulated over a long period are capable of providing big data support for understanding students’ learning needs. Students should be given daily or weekly feedback on their skill mastery. It is dangerous to use only the final grades as the evaluation criterion because it is very difficult to define a clear and fair standard for any given skill. It is also believed that the final grades can also be very daunting and cause stress to both students and teachers. With cognitive diagnosis theory, a number of clearly defined skills, along with the strengths and weaknesses of students’ knowledge, help students understand why they are doing the exercises and how much they are willing to practice for improvement. In addition, it is very important to provide timely feedback to students and/or teachers so they can effectively improve their learning and/or instruction efficiency. If a student is asked to set goals in the far future or receives feedback only after several days, weeks, or even months, the student cannot ‘connect the dots’ between what he or she has done and the provided feedback. Timely and constant feedback is very motivating because it shows that the work you have done today improves your skills and that the teacher or other students have noticed your efforts. On the other hand, the accumulation of teaching diagnostic data provide an opportunity for further analysis and exploration of data from multiple tests. This provides the possibility of identifying the fundamental characteristics and problems during the entire learning period, as well as the strengths and weaknesses of individual thinking style. In this way, students can understand themselves more clearly, and their personalities can be fully developed. As a result, the dominant position of students in their learning is highlighted and strengthened. Especially, students’ awareness of their dominant position is enhanced, and then, they will endeavor to maintain this position during learning, playing the role of learners.

Recorded information and feedback on the learning process via the CLDS help teachers evaluate students from a development perspective. In order to achieve individualized instruction, teachers should give students more support or more challenging tasks individually, because all the students have different levels of skills and knowledge, and they learn at their own pace and are motivated by different things. If an advanced student does not receive more challenging tasks, he/she will easily feel boring. If a student falls behind in a strictly regulated course, this might lead to feelings of unworthiness and willingness to stop trying. With the help of the CLDS, teachers will better understand their students’ individualized needs, the performance characteristics of different groups, and the learning situation of the entire class. If teachers observe and provide feedback regularly, they will notice that some students have problems with certain skills and then be able to immediately provide more support or easier tasks to keep those students motivated and progressing. Teachers can even notice when students have acquired the required skill level of the course and, thus, allow them to move forward in their studies.

The CLDS can be expanded for further applications, and its value is expected to increase along with the growing number of users. As time goes by and more schools are using the CLDS, the system accumulates more testing data of students and more exam items. Therefore, local item banks of schools are being established during the process. These local item banks contain not only item parameters as in traditional item banks but also attribute identification for each item based on cognitive diagnosis. Additionally, the item banks include historical responses previously made by students, typical types of wrong answers, and teachers’ comments. The combination of local item banks with the CLDS will greatly reduce the overall burden on teachers and improve the efficiency of the entire testing process, thus effectively promoting the evidence-based reform in education and the improvement of teaching quality. Moreover, the establishment of local item banks further expands the function of the CLDS. With the help of the CLDS, teachers can assign individualized homework to students, and students can carry out adaptive learning and exercises. Based on the adaptive algorithm, the CLDS recommends targeted homework assignments to different students based on their test results, ensuring that students can address their weaknesses through practice.

Over the last few decades, there has been a progressive change in education toward more student-centered and self-directed learning (Johnson et al., 2012). Increasingly, information and communications technology (ICT) enables individualized learning by offering students greater diversity in their learning and more flexible and personalized learning spaces (Brown and Green, 2015). So far, there is still room for improvement in selecting student-centered learning resources and providing individualized data. There are plans to integrate the CLDS with other online education resources (e.g., digital education platforms) to provide more content for promoting students’ independent learning. Furthermore, data obtained from various tests for each test taker pose challenges to statistical analysis. How to adopt appropriate statistical analysis models to further explore the characteristics and types of students’ personalized learning is a meaning issue to be investigated.

### The Improvement Direction of CLDS

One of the advantages of CLDS is that the analysis and feedback are based on the cognitive diagnosis model. From this perspective, the selection of the appropriate psychometric model is the bedrock of CLDS. Researchers have found that the cognitive diagnostic results generated by the DINA model are consistent with those obtained from an academic achievement test that the students took previously (Liu H. et al., 2013). The results of the DINA model possess face validity and are easy to interpret. Besides, the feedback is easy for teachers and students to understand. Therefore, we chose the DINA model in our system for both theoretical and practical considerations. However, even though the DINA model worked well in the current study, many other models should be included in future studies.

Further considerations should be given to how cognitive attributes interact to arrive at an item response, as well as other compensation models that describe the mutual compensation relationship of attributes. Several other models may generate better results, such as the fusion model (Hartz et al., 2002), the noisy input deterministic and gate (NIDA) model (Maris, 1999), the hierarchical DINA model (de la Torre and Douglas, 2004), the deterministic input noisy or gate (DINO) model (Templin and Henson, 2006), and the multicomponent latent trait model (Embretson, 1985).

The psychometric model of the CLDS assumes binary classifications (mastery or nonmastery) for attributes, which often fails to meet the needs of users. The probability of mastery is reported in the current report to provide more information, but the interpretation is difficult and always misleading (Bradshaw and Levy, 2019). In practice, partial or incomplete mastery is possible, and the heterogeneity in response data may not be well explained by the model we use. Therefore, a more flexible cognitive diagnosis model, which allows for partial mastery (Shang, 2019), should be considered further.

The CDM used in the CLDS is a single-level model, not taking into account the nested structure of data of students. However, this data structure is popular in educational assessment (Goldstein, 2010). Recently, there have been some attempts to extend single-level CDMs to multilevel ones (e.g., von Davier, 2010; Wang and Qiu, 2019). The results indicated that the estimates of item parameters and their standard errors were not affected, but ignoring multilevel structures would result in a poorer recovery of individual latent profiles (Wang and Qiu, 2019). Therefore, in order to obtain more accurate diagnosis results, more appropriate multilevel CDMs should be considered in the future system improvement process.

In applications, the latent Q-matrix, which is often constructed by experts, is subjective and thus can be misspecified. The misspecification of the Q-matrix may lead to inaccurate inferences on the latent attribute profiles. Moreover, in practice, we found it difficult for teachers to identify the attributes of newly designed items, which frustrated teachers’ motivations to apply the system. Although CLDS allows teachers to update the Q-matrix manually, it is worthwhile studying how to automatically detect and update the Q-matrix based on the response data, thus reducing the subjectivity of Q-matrix specification and making the system more friendly. In the literature, researchers have been developing methods to estimate the Q-matrix from the response data (e.g., DeCarlo, 2012; Chiu, 2013; Liu J. et al., 2013; Chen et al., 2015; de la Torre and Chiu, 2016). More recently, Xu and Shang (2018) proposed a stepwise estimation method to update the Q-matrix based on the idea of statistical learning. Both simulation studies and case studies support that Shang’s method can detect most of the misspecified items. Therefore, how to integrate these new methods into CLDS is a challenging and exciting issue in applications, which can be explored in the future.

## Data Availability

The datasets generated for this study are available on request to the corresponding authors.

## Ethics Statement

The study was reviewed and approved by Institutional Review Board of the Faculty of Psychology in Beijing Normal University, as well as by the committee in local government. All school teachers, students and their parents were provided with written informed consent. Therefore, the school teachers, students, and their parents had clear understanding about this project and how the data were collected.

## Author Contributions

XY and HL designed the research, analyzed the data, and wrote the manuscript. ML analyzed the data and wrote the manuscript. YX wrote the manuscript.

## Conflict of Interest Statement

The authors declare that the research was conducted in the absence of any commercial or financial relationships that could be construed as a potential conflict of interest.

## Footnotes

1 http://www.biyouxue.com/
